# Effects of a 12-week resistance and aerobic exercise program on muscular strength and quality of life in breast cancer survivors: Study protocol for the EFICAN randomized controlled trial

**DOI:** 10.1097/MD.0000000000017625

**Published:** 2019-11-01

**Authors:** Alberto Soriano-Maldonado, Álvaro Carrera-Ruiz, David M. Díez-Fernández, Alba Esteban-Simón, Mercedes Maldonado-Quesada, Nuria Moreno-Poza, María del Mar García-Martínez, Celia Alcaraz-García, Rosa Vázquez-Sousa, Herminia Moreno-Martos, Antonio Toro-de-Federico, Nur Hachem-Salas, Eva Artés-Rodríguez, Manuel A. Rodríguez-Pérez, Antonio J. Casimiro-Andújar

**Affiliations:** a Department of Education, Faculty of Education Sciences; b SPORT Research Group (CTS-1024), CERNEP Research Centre, University of Almería; c Hospital Universitario Torrecárdenas, Servicio de Radiodiagnóstico, Unidad de Mama, Almería, Spain; d Servicio Andaluz de Salud, Unidad de Gestión Clínica Almería Periferia; e Servicio Andaluz de Salud, Unidad de Gestión Clínica Ciudad Jardín; f Servicio Andaluz de Salud, Unidad de Gestión Clínica Mediterráneo-Torrecárdenas, Distrito Sanitario; g Area of Statistics and Operative Research, Department of Mathematics, Faculty of Sciences, University of Almería, Almería, Spain.

**Keywords:** breast cancer, cancer-related fatigue, exercise, health-related quality of life, muscular strength, resistance training

## Abstract

**Background:**

The number of people living with the side effects of breast cancer treatment (eg, loss of muscular mass and muscular strength, upper-limb mobility and disability, lymphedema, cardiac toxicity, and reduced quality of life) is increasing yearly. These consequences can be improved through exercise, specially combining resistance and aerobic training. Previous exercise trials have not been consistent in applying training principles and standardized reporting, and this partly explains the variability in obtained results. The aim of this study is to assess the effect of a 12-week supervised resistance exercise program combined with home-based aerobic exercise, compared with home-based aerobic exercise only, on muscular strength and several aspects of health-related quality of life in breast cancer survivors. To maximize transparency, replicability, and clinical applicability, the intervention is described following the consensus on exercise reporting template.

**Methods:**

This study is a parallel-group randomized controlled trial in which 60 female breast cancer survivors, who have completed central treatments of the disease in the last 5 years, will be randomly assigned to either an experimental group that will perform a total of 24 progressive resistance training sessions for 12 weeks (ie, 2 weeks of individual training and 10 weeks of micro-group training) and will be requested to undertake 10,000 steps/d, or a control group that will be requested to undertake 10,000 steps/d, only. Outcomes will be evaluated at baseline and at week 12. Primary outcome measure is peak isometric muscular strength of the lower- and upper-body, assessed with several exercises through an electromechanical dynamometer. Secondary outcomes include cardiorespiratory fitness, upper-joint mobility and disability, health-related quality of life, cancer-related fatigue, depression, life satisfaction, and presence of lymphedema.

**Discussion:**

This study aims to investigate the extent to which a 12-week supervised and progressive resistance exercise program, in addition to home-based aerobic physical activity, might improve muscular strength and health-related quality of life in breast cancer survivors. The comprehensive description of the intervention will likely contribute to enhancing exercise prescription in this population.

**Trial registration number:**

ISRCTN14601208.

## 1 Introduction

In women, breast cancer is the most commonly diagnosed type of cancer (ie, ∼2.1 million new cases every year) and the leading cause of death worldwide.^[1]^ The incidence of breast cancer in Spain is increasing. While there were approximately 26,000 new diagnoses in 2017, recent estimations anticipate over 32,000 diagnoses in 2019.^[2]^ In the city of Almería (ie, southern Spain) it is estimated that approximately 500 new cases appear yearly.^[3]^ Nevertheless, ongoing advances in early detection and treatment of breast cancer have led to a significant mortality reduction. For instance, survival following breast cancer, in a developed country such as Canada, has been reported to be of 87%.^[4]^ In Europe, breast cancer mortality is estimated to be reduced over 10% by 2020,^[5]^ with the exception of Spain, as Spain has the lowest mortality rate in Europe.^[5]^

The above-mentioned breast cancer mortality reduction over time implies that a yearly increasing population is living long after cancer diagnosis and treatment, thus facing many short-, mid-, and long-term side effects. Therefore, research addressing the management of the breast cancer-related side effects is of major clinical and public health relevance. Common side effects following breast cancer include the presence of lymphedema,^[6]^ cardiac toxicity,^[7]^ depression,^[8]^ fatigue,^[9]^ bone health issues, or obesity.^[10]^ These problems, together with a significant loss of upper limb mobility^[11,12]^ and a major loss of muscular strength and muscle mass, compromise life satisfaction and quality of life^[13]^ and are to be monitored in the follow-up of breast cancer.^[14]^ In particular, muscular strength during treatment has been reported to be 25% lower in lower extremities and 12% to 16% lower in upper extremities compared to healthy individuals,^[15]^ and similar trends seem to occur regarding cardiorespiratory fitness^[16,17]^ and upper limb function and mobility.^[11]^ It is important to note that this tendency might worsen in the absence of physical exercise following treatment.

Current guidelines for the management of breast cancer survivors include health promotion counseling related to physical activity.^[14]^ In this line, for ethical reasons, any exercise-based clinical trial should ensure that all trial participants, at least, meet the international physical activity guidelines (ie, at least 150 minutes per week^[18]^). In addition, structured physical exercise is an attractive option in the follow-up of breast cancer as it might counteract or, at least, benefit, several (if not all) of the above-mentioned side effects of breast cancer.^[19–22]^ In particular, resistance training has shown to enhance muscular function and body composition and, to some extent, fatigue, not only during treatment, but also in the long-term follow-up,^[23]^ and it is safe for limb-related issues such as lymphedema.^[24–27]^ Resistance training at least once a week has shown to reduce the mortality risk by 33% in breast cancer survivors.^[28]^

However, description of exercise interventions in exercise-based clinical trials, including breast cancer trials,^[29]^ has been consistently poor.^[30]^ In their recent systematic literature review, Neil-Sztramko et al^[29]^ concluded that “no studies of exercise in women with breast cancer attended to all principles of exercise training or reported all components of the exercise prescription in the methods, or adherence to the prescription in the results,” which precludes transparency and replicability of clinical trials in this population. The systematic review of Fairman et al^[31]^ revealed that resistance training prescription is very heterogeneous across studies and largely underdeveloped. Failure to apply the exercise principles may explain the heterogeneity observed across study outcomes in various systematic reviews and meta-analyses.^[20,32–36]^ This opens a window of opportunity for upcoming trials to correctly implement the exercise principles and provide comprehensive details of interventions,^[37]^ thus contributing to the development of future resistance training guidelines in breast cancer survivors.^[29]^

The primary aim of this study is to assess the effect of a 12-week supervised resistance exercise program combined with home-based aerobic training, compared with home-based aerobic training only, on muscular strength in breast cancer survivors. Secondary aims are to assess the effects of the intervention on cardiorespiratory fitness, upper-limb mobility and disability, health-related quality of life, cancer-related fatigue, depressive symptoms, life satisfaction, body composition, and lymphedema in this population.

## 2 Material and methods

### 2.1 Design and protocol registration

The EFICAN (Ejercicio FÍsico para pacientes con CÁNcer de mama) study is a parallel-group randomized controlled trial registered at the ISRCTN registry (ISRCTN14601208) on August 1, 2019, before the enrolment of participants begun (ie, on August 12, 2019).

### 2.2 Setting and eligibility criteria

Participants have been recruited through local associations and advertisements in local newspapers, radio, and social media including social networks, and through referral from clinical oncologists from the Torrecárdenas University Hospital. Eligible participants are voluntary women aged 18 to 65 who have undergone breast cancer surgery and have finished core treatments (ie, chemotherapy and/or radiotherapy) in the past 5 years. The exclusion criteria are defined as follows:

(1) presenting with metastatic breast cancer,
(2) awaiting breast reconstruction in the following 6 months,
(3) presenting with any pathology that might prevent participants from exercising (ie, decompensated heart failure, unstable ischemic heart disease, severe untreated high blood pressure, moderate-severe valvopathies, aortic aneurysm, moderate-severe chronic obstructive pulmonary disease, pulmonary hypertension, chronic respiratory insufficiency), and
(4) regularly performing >300 minutes per week of structured exercise. This study has been reviewed and approved by the Ethics Committee of the Torrecárdenas University Hospital, Almería, Spain (ref: Ejercicio-CáncerUAL[98/2019]), on July 31, 2019.

### 2.3 Procedures

All potential voluntary participants fill out an online form indicating basic information about their disease, years from treatment termination, and so on. Potential participants who are eligible based on the form information attend a personal screening with our Medical Doctors (MD) at public health care centers. The MD conduct an initial screening and revise the clinical records to confirm that all inclusion criteria are met and that participants are able to undertake physical exercise, in case of being randomized to such group. The MD are also responsible for obtaining participant's informed consent. Subsequently, the participants who meet inclusion criteria attend the exercise laboratory at University of Almería to undertake the physical examination. This study follows the SPIRIT guidelines for randomized trials protocols.^[38]^ The funding source has no role in the study. All databases including personal information will be collected by the principal investigators (AS-M and AJC-A) only, who will be responsible for protecting confidentiality.

### 2.4 Sample size

The sample size was calculated for the primary outcome (ie, muscular strength) using Stata v.13 (StataCorp LP, College Station, TX). Based on the results of the meta-analysis by Strasser et al,^[23]^ a difference in the change between groups in lower limb muscle strength of at least 6.9 kg would be clinically relevant. Assuming a common standard deviation of 7.5 kg, a 90% power, an alpha error of 5%, and considering a potential dropout of 20%, we will aim at enrolling 60 participants.

### 2.5 Randomization, treatment allocation, and blinding

After baseline assessment, each participant will be randomized to either an experimental group (EG) or a waiting-list control group (CG). Before participant's recruitment, a blinded investigator (AS-M) created a computer-generated simple randomization sequence, to allocate participants in either group. Individual allocations were held in sealed, opaque, and consecutively numbered envelopes. A member of the staff not involved in the trial assessment or interventions will open the envelopes in front of participants and assign them to the groups.

The data analysts and the primary outcome assessors will be blinded to the participant allocation. However, due to the nature of the intervention (ie, supervised resistance exercise), the participants will be aware of the group they are allocated to.

### 2.6 Intervention

Besides the intervention described below, all participants are requested to continue their normal life and continue their treatment (ie, medication) during the trial. The intervention period starts on September 23, 2019, and ends on December 13, 2019.

#### 2.6.1 Experimental group

To maximize transparency and replicability of the exercise protocol, we followed the guidelines stated on the Consensus on Exercise Reporting Template (CERT; Table S1).^[37]^ Participants assigned to the intervention group will carry out 2 strength training sessions weekly for 12 weeks. These 12 weeks are divided into 2 phases: a first phase of individual training (ie, 1:1 ratio) with a duration of 2 weeks and a second phase of 10 weeks in which group (ie, 4–6 participants) workouts are performed. Therefore, the program comprises a total of 24 training sessions (ie, of 60 minutes duration). In addition, the participants will be requested to undertake home-based aerobic training consisting in meeting the physical activity guidelines of 10,000 steps per day.^[39,40]^ The compliance with this requirement will be monitored through the Z11 Smart Band (Smart&Sport, Shenzhen, China) activity bracelet. The supervised training sessions will be led by exercise professionals (i.e. personal trainers; PT) with a degree in Physical Activity and Sport Sciences and with specific training in exercise for breast cancer patients. The exercise program will be carried out in a fitness room at the Almeria town hall (400 m^2^), which is equipped with aerobic and resistance training areas. The detailed description of the exercise equipment to be used during the training program is presented as additional file (Table S2).

Table 1 summarizes the periodization of the resistance training program. During phase 1 (ie, weeks 1 and 2; individual sessions), each participant will perform a total of 4 sessions in which the exercise professional will determine individual needs and limitations (eg, shoulder mobility, pain, etc) and participants will learn basic movement patterns that will be required in the following phase. During phase 2 (ie, weeks 3–12; group sessions), the participants will undertake circuit training including mobility and stability exercises and resistance training. There will be several stations in which participants will perform an exercise before moving to the next station. The starting level of each participant will be set considering their baseline muscular strength and the work undertaken in weeks 1 and 2.

**Table 1:**
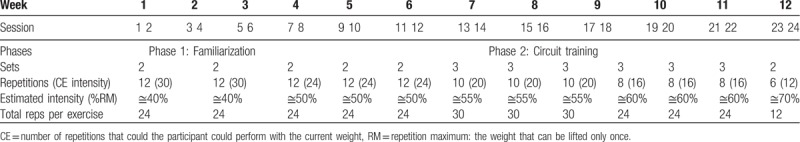
Periodization of the supervised resistance training program.

Each supervised exercise session consists of 3 parts. Part 1 is a preparatory part of approximately 15-20 minutes, divided into 5 minutes of low-intensity aerobic activity (50%–65% of the heart rate reserve) either on a treadmill o an elliptical trainer, 2 thoracic mobility exercises and 2 central stability (CORE) exercises (5-8 minutes), 2 scapulohumeral joint stability and 2 dynamic stability exercises (5-8 minutes). The exercise intensity for part 1 will be 3/10 quantified through the OMNI perceived exertion scale for resistance exercise (OMNI-RES).^[41]^ Part 2 (ie, the main part) comprises a circuit of 4 dynamic resistance exercises (ie, bilateral dead lift, bilateral seated row, bilateral squat, and bilateral seated bench press). Resistance training intensity will be equivalent to 40% to 70% of 1 repetition maximum (1 RM) and will be individually calculated so that participants progressively work with a training load that can be lifted 24 times (ie, 24 RM) to a training load that can be lifted 12 times (12 RM). Progressions will generally occur weekly. Although 60% to 70% of 1 RM is recommended in healthy adults to improve strength,^[42]^ previous research has shown that more moderate intensities (ie, 40%–60% of 1 RM) can improve muscular power, strength, muscular size, and functional tasks even in older people.^[43]^ Exercise intensity will be individually quantified through the character of the effort (CE; which represents the number of repetitions actually performed out of the maximum number of repetitions that could be performed with a given load), as previously reported,^[44,45]^ and participants will be asked to report their subjective level of effort (ie, after each exercise) through the OMNI-RES.^[41]^ The CE will be distant from muscle failure to maximize strength^[46]^ gains^[47]^ and minimize risks. According to previous research,^[48,49]^ in each set, participants will perform half of the maximum number of possible repetitions to achieve greater mean repetition velocity, lower impairment of neuromuscular performance, faster recovery, reduced hormonal response and muscle damage, and lower reduction in heart rate variability.^[48,49]^ For greater strength gains, participants will be advised to perform the concentric phase of each exercise at maximum voluntary velocity.^[43,50]^ The resting periods between sets of the same exercise will be of approximately 3 minutes as recommended in older adults.^[43]^ Part 3 consists of a collective calm-down, including dynamic/static stretching of major muscle groups (ie, pectoralis major, dorsal width, quadriceps, hamstrings), and a general group evaluation of the session (Table S3). The main exercises that are to be performed along the exercise program, divided by part of the session, are presented in Table 2.

**Table 2:**
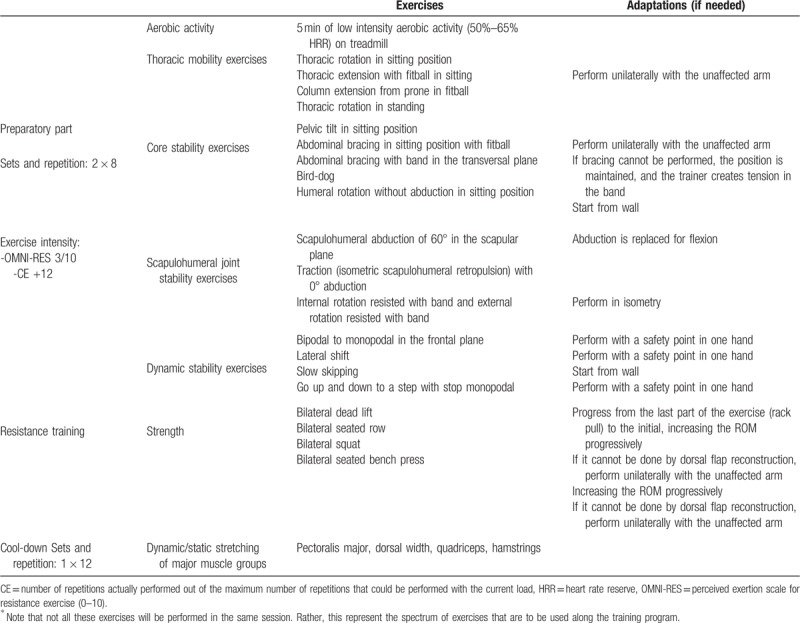
Exercises that are to be performed along the resistance training program^∗^.

All participants will be instructed to report any difficulties, limitations or needs so that the intervention can be adapted to individual needs. To maximize adherence, participants will be sent motivational messages (ie, either through WhatsApp or email) every 2 weeks, and participants who miss sessions will be contacted to ask for reasons and reallocate the schedule to undertake the session another day. Adherence to the program will be reported as the median attendance frequency and the proportion of patients attending ≥75% (ie, 18 sessions; the minimum pre-defined attendance to assess efficacy). Adherence to the exercise program, potential adverse events, and other relevant information will be measured throughout the whole intervention period using a comprehensive tally sheet to be completed daily by the PT during and after each training session. The detailed tally sheet to be used in this study, based on a previous well-designed exercise protocol,^[51]^ is shown as additional file (Table S3). There will not be any nonexercise component in this intervention.

#### 2.6.2 Control group

Participants assigned to the CG will be required to undertake aerobic activity by performing a minimum of 10,000 steps per day to meet the physical activity guidelines but will not be involved in supervised resistance training. We thus aim to guarantee that all enrolled participants benefit from being physically active as they are all required to comply with aerobic activity recommendations. Compliance with the step goal will be monitored through the Z11 Smart Band (Smart&Sport, Shenzhen, China) activity bracelet in the same way as the EG. For ethical reasons, the CG will have the possibility to undertake the resistance exercise intervention once the trial finalizes.

### 2.7 Outcome measures

All outcome measures will be assessed at baseline and at week 12 (ie, after concluding the intervention period). Baseline assessment will be carried out during the 14 days before the beginning of the intervention and follow-up assessments will be conducted in the following 10 days from finalizing the intervention. The principal investigators (AS-M and AJC-A) are responsible for the dataset and will only distribute it between trial investigators who are included in the funded project.

#### 2.7.1 Primary outcome measure: muscular strength

The primary outcome is muscular strength because of its major clinical relevance, as it is key for a person to undertake daily activities like working, housework, and dressing among many others. Muscular strength will mainly be assessed with an electromechanical dynamometer (Dynasystem Research, Symotech, Granada, Spain)^[52–54]^ and measured in N. The primary outcome measures will be the following:

##### 2.7.1.1 Upper-body muscular strength

**Figure 1:**
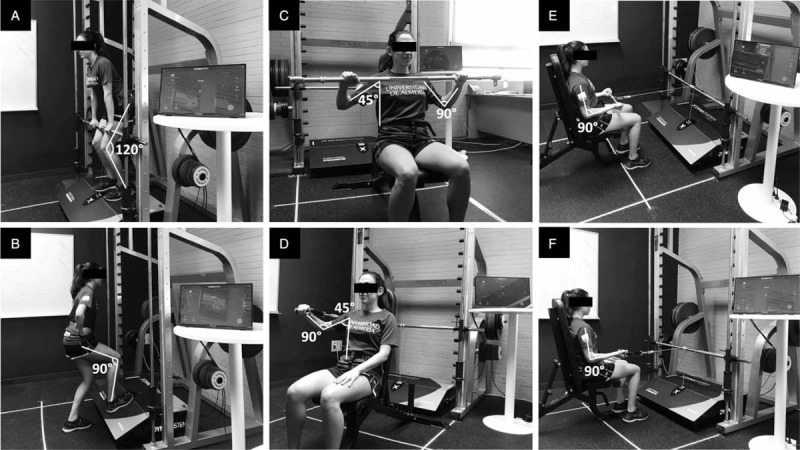
Assessment muscular strength (A) mid-thigh isometric pull test and upper-body muscular strength, (B) unilateral isometric knee extension in closed kinetic chain at 90°, (C) bilateral isometric seated bench press, (D) unilateral isometric seated bench press, (E) bilateral isometric seated row, (F) unilateral isometric seated row.

Assessed as the average of the standardized score (*z*-score = [value−mean]/standard deviation) of 2 different exercise tests, including the sum of right and left unilateral isometric seated bench press (Fig. 1D), and the sum of right and left unilateral isometric seated row (Fig. 1F).

##### 2.7.1.2 Lower-body muscular strength

Assessed as the average of the standardized score (*z*-score = [value-mean]/standard deviation) of 2 different exercise tests, including the sum of right and left unilateral isometric knee extension in closed kinetic chain at 90° (average of the right and left knees; Fig. 1B), and the mid-thigh isometric pull test (ie, bilateral knee and hip extension; Fig. 2A).

##### 2.7.1.3 Overall muscular strength

Standardized score computed as the average of the normalized score (*z*-score = [value-mean]/standard deviation) of all the above-mentioned upper- and lower-body exercise tests (Fig. 1).

#### 2.7.2 Secondary outcomes

##### 2.7.2.1 Other muscular strength variables

- Bilateral isometric seated bench press (Fig. 1C).
- Isometric seated bench press bilateral deficit [bilateral deficit = (100 × bilateral/(right unilateral + left unilateral)) − 100].
- Bilateral isometric seated row (Fig. 1E).
- Isometric seated row bilateral deficit [bilateral deficit = (100 × bilateral/(right unilateral + left unilateral)) − 100].
- Handgrip strength (of the right and left sides) assessed with a digital dynamometer (Model T.K.K.540; Takei Scientific Instruments Co, Ltd, Niigata, Japan). Within each side, the highest score of 2 trials will be considered for the analyses.
- The difference between right unilateral and left unilateral handgrip strength, assessed with a digital dynamometer (Model T.K.K.540; Takei Scientific Instruments Co, Ltd).

##### 2.7.2.2 Cardiorespiratory fitness

Cardiorespiratory fitness (ie, maximum oxygen consumption [VO_2máx_]) will be estimated through the Siconolfi Step Test. This test has been developed for use in epidemiological studies^[55]^ and has been used in different populations.^[55–58]^ The test is performed on a wooden box (25.4 cm × 30.5 cm × 45.7 cm) made ad hoc. Heart rate (HR) is monitored using a HR monitor (Polar V800; Polar Electro, Kempele, Finland). In stage 1, the participant has to step up and down the box at a frequency of 17 times per minute for 3 minutes. If the average HR of the participant during the last 30 seconds of stage 1 reaches or exceeds 65% of the estimated maximal heart rate (HRmax), the test finalizes. Otherwise, the participant rests for one minute and undetakes stage 2, increasing the step frequency to 26 times per minute for 3 minutes. If the HR criteria is not met in the last 30 seconds of stage 2, stage 3 takes place increasing the step frequency to 32 times per minute for 3 minutes. In this way, the VO_2máx_ is estimated using the following expression^[55,59]^:
 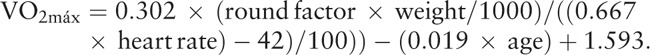


According to the original protocol, if 65% of the estimated HRmax is reached or exceeded during the stage 1, the round factor is 16.287, whereas the round factor is 24.910 and 35.533 if the 65% of the estimated HRmax is reached in stage 2 or 3, respectively.

##### 2.7.2.3 Shoulder mobility

The range of shoulder flexion in supine position will be assessed through digital goniometry (HALO Digital Goniometer; HALO Medical Devices HQ, Sydney, Australia) as reported elsewhere.^[60]^

##### 2.7.2.4 Physical disability of the upper limb

Physical disability of the upper limb will be assessed with the Spanish version^[61]^ of the Disabilities of the Arm, Shoulder, and Hand (DASH) questionnaire.^[62]^

##### 2.7.2.5 Cancer-related quality of life

Cancer-related quality of life will be assessed with the Spanish version^[63]^ of the European Organization for Research and Treatment of Cancer Quality of Life Questionnaires-Core30 (EORTC QOL-30),^[64]^ including the Spanish version^[65]^ of the extension for breast cancer (EORTC QLQ-BR23).^[66]^ The global score ranges from 0 to 100 where higher scores indicate better quality of life. Cancer specific quality of life will also be assessed with the Spanish version^[67]^ of the Functional Assessment of Cancer Therapy-Breast (FACT-B)^[68]^. The global score ranges from 0 to 148 where higher scores indicate better quality of life.

##### 2.7.2.6 Cancer-related fatigue

Cancer-related fatigue will be assessed with the Spanish version^[69]^ of the Functional Assessment of Cancer Therapy-Fatigue (FACT-F)^[70]^ questionnaire.

##### 2.7.2.7 Depressive symptoms

Depressive symptoms will be assessed with the Spanish version^[71]^ of the 20-item for Epidemiologic Studies-Depression Scale (CES-D)^[72]^

##### 2.7.2.8 Life satisfaction

Life satisfaction will be assessed with the Spanish version^[73]^ of the satisfaction with life scale (SWLS).^[74]^

##### 2.7.2.9 Anthropometrics and body composition

Weight (in kg) and body composition (including body fat percentage, fat-free mass [kg], etc) will be assessed with a bioelectrical impedance device (InBody 120; InBody Co Ltd, Seoul, Korea) and height will be measured with a digital stadiometer (Seca 202; Seca Ltd, Hamburg, Germany). Hip and waist circumference will be measured with an anthropometric tape (Seca 201; Seca Ltd). Body mass index (kg/m^2^), waist-to-height ratio, and waist-to-hip ratio, will be calculated.

##### 2.7.2.10 Arm volume and lymphedema

Arm volume and lymphedema will be evaluated by measuring the perimeter of the arm at different points that will be 6 cm apart, to estimate the volume of both arms using the following expression^[75]^:
 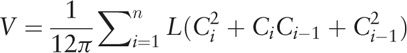


where n = number of segments, *L* = length of each segment, and *C*_*i*_ and *C*_*i*−1_ = circumference at each end of the segment. Subsequently, both volumes will be compared, and the presence of lymphedema will be considered when the volume of the affected arm exceeds the volume of the non-affected arm by 3-10%.^[76–78]^

### 2.8 Data collection procedure

Baseline (ie, from September 9 to September 20, 2019) and follow-up (from December 16 to December 20, 2019) evaluations will be performed at the Exercise Laboratory of the University of Almería. Each participant begins filling out online questionnaires including health-related quality of life, cancer-related fatigue, depression, life satisfaction, and a general sociodemographic questionnaire. Thereafter, body composition is assessed in a private room, followed by shoulder joint mobility and presence of lymphedema, upper- and lower-body muscular strength and cardiorespiratory fitness.

### 2.9 Statistical analysis

The distribution of the main study variables will be assessed through histogram and Q-Q plots. Descriptive characteristics will be presented using the mean and standard deviation for normally distributed quantitative variables, the median and interquartile range for non-normally distributed quantitative variables, and the number and frequency for categorical variables. The comparability of the groups will be checked at baseline and potential confounders that present baseline group differences will be included in the analyses. The between-group differences in the primary and secondary outcomes will be assessed through linear or quantile regression, as appropriate. As we aim at assessing efficacy, the primary analyses are defined as per-protocol, where participants from the exercise group will be included if their attendance to the exercise sessions is ≥75%. Sensitivity analyses will also be carried out using a minimum attendance of ≥90% and using the intention-to-treat principle. Blinded investigators (AS-M and EA-R) will handle all hypothesis testing. The main analyses will be conducted with Stata v.13.1 (StataCorp LP, College Station, TX). Statistical significance will be set at *P* < .05.

## 3 Discussion

This study will examine the effects of a 12-week supervised resistance exercise program combined with home-based aerobic training, compared with home-based aerobic training only, on muscular strength and health-related quality of life, among other relevant outcomes that are compromised during the treatment follow-up in breast cancer survivors. A number of relevant publications have highlighted that the poor description^[31]^ of exercise programs and the scarce application of training principles in breast cancer clinical trials^[29]^ might explain the inconclusive results in several relevant domains^[36]^ regarding common side-effects of treatment. This study aims at overcoming this relevant issue by presenting a comprehensive description of the supervised resistance exercise intervention that can be replicated and applied in clinical or other settings. Importantly, we follow the CERT guidelines, which should be implemented in all health-related exercise trials. This trial will determine the extent to which a supervised resistance training twice per week improves muscular strength and several relevant outcomes related to quality of life in breast cancer survivors, provided all participants complete the minimum amount of aerobic activity (ie, defined as achieving a minimum of 10,000 steps per day). The results of this relatively small trial will likely be useful for the development of future exercise prescription^[31]^ for breast cancer survivors.

### 3.1 Study limitations

This study has limitations that must be underlined. First, adherence to the exercise programs is a challenge, and several adherence strategies have been incorporated to maximize attendance and motivation. Only women will be enrolled in this study. External validity will not be ensured due to potential selection bias (ie, patients who volunteer to participate may also be the most motivated to exercise). However, the reasons for non-participation and abandonment will be collected and reported.

## Acknowledgments

The authors would like to thank the associations of patients that have provided support in the recruitment process, including “Asociación Proyecto Mariposa,” “Asociación Española Contra el Cáncer,” and “Asociación Amama Almería.”

The Instituto Profesional Ejercicio Físico y Cáncer *(IPEFC)* is also gratefully acknowledged for providing training and support to the sports science professionals involved in the intervention, and for its contribution to the design of the intervention program. The owners of Dynasystem (Dynasystem Research, Symotech, Granada, Spain) are also gratefully acknowledged, for allowing us to use their materials in the present study.

The authors would also like to acknowledge Mr. Manuel Martín Olvera, Mr. Carlos Lloret Michán, Mrs. Carolina González Fernández, Dr. Antonio Orta Cantón, Dr. Miguel Lorenzo Campos, Dr. Mariana Teresa Peña Perea, and Mrs. Nereida Padilla Asensi for their support during the study design, recruitment, and implementation of the study.

## Author contributions

**Conceptualization:** Alberto Soriano-Maldonado, Rosa Vázquez-Sousa, Manuel A. Rodríguez-Pérez, Antonio J. Casimiro-Andújar.

**Formal analysis:** Alberto Soriano-Maldonado, Antonio J. Casimiro-Andújar.

**Funding acquisition:** Alberto Soriano-Maldonado, Antonio J. Casimiro-Andújar.

**Investigation:** Alberto Soriano-Maldonado, Álvaro Carrera-Ruiz, David M. Díez-Fernández, Alba Esteban-Simón, Mercedes Maldonado-Quesada, Nuria Moreno-Poza, María del Mar García-Martínez, Celia Alcaraz-García, Rosa Vázquez-Sousa, Herminia Moreno-Martos, Antonio Toro-de-Federico, Nur Hachem-Salas, Eva Artés-Rodríguez, Manuel A. Rodríguez-Pérez, Antonio J. Casimiro-Andújar.

**Methodology:** Alberto Soriano-Maldonado, Manuel Rodríguez-Pérez, Eva Artés-Rodríguez, Antonio J. Casimiro-Andújar.

**Project administration:** Alberto Soriano-Maldonado, Antonio J. Casimiro-Andújar.

**Resources:** Alberto Soriano-Maldonado, Antonio J. Casimiro-Andújar, Rosa Vázquez-Sousa, Herminia Moreno-Martos, Antonio Toro-de-Federico, Nur Hachem-Salas.

**Software:** Alberto Soriano-Maldonado, Eva Artés-Rodríguez.

**Supervision:** Alberto Soriano-Maldonado, Antonio J. Casimiro-Andújar.

**Validation:** Eva Artés-Rodríguez.

**Visualization:** Alberto Soriano-Maldonado, David M. Díez-Fernández, Alba Esteban-Simón, Mercedes Maldonado-Quesada, Nuria Moreno-Poza, María del Mar García-Martínez, Celia Alcaraz-García.

**Writing – original draft:** Alberto Soriano-Maldonado, David M. Díez-Fernández, Manuel A. Rodríguez-Pérez.

**Writing – review and editing:** Alberto Soriano-Maldonado, Álvaro Carrera-Ruiz, David M. Díez-Fernández, Alba Esteban-Simón, Mercedes Maldonado-Quesada, Nuria Moreno-Poza, María del Mar García-Martínez, Celia Alcaraz-García, Rosa Vázquez-Sousa, Herminia Moreno-Martos, Antonio Toro-de-Federico, Nur Hachem-Salas, Eva Artés-Rodríguez, Manuel A. Rodríguez-Pérez, Antonio J. Casimiro-Andújar.

Alberto Soriano-Maldonado orcid: 0000-0002-4626-420X.
