# Effects of a 12-week resistance and aerobic exercise program on muscular strength and quality of life in breast cancer survivors: Study protocol for the EFICAN randomized controlled trial: Erratum

**DOI:** 10.1097/MD.0000000000018419

**Published:** 2019-12-10

In the article, “Effects of a 12-week resistance and aerobic exercise program on muscular strength and quality of life in breast cancer survivors: Study protocol for the EFICAN randomized controlled trial”,^[1]^ which appears in Volume 98, Issue 44 of *Medicine*, the table headers for Table 1 were misleading and showed phase 1 extending into week 3. The correct table is:

**Table 1:**
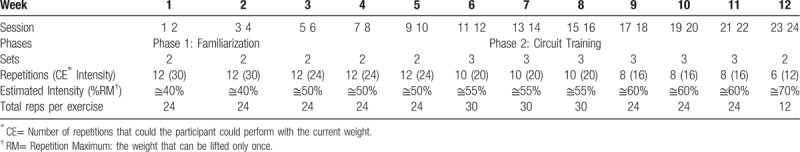
Periodization of the supervised resistance training program.
